# New Advances in Attention-Deficit/Hyperactivity Disorder-like Dogs

**DOI:** 10.3390/ani14142067

**Published:** 2024-07-14

**Authors:** Ángela González-Martínez, Susana Muñiz de Miguel, Francisco Javier Diéguez

**Affiliations:** 1 Veterinary Teaching Hospital Rof Codina, Santiago de Compostela University, 27002 Lugo, Spain; 2 Anatomy, Animal Production and Clinical Veterinary Sciences Departament, Santiago de Compostela University, 27002 Lugo, Spain; susana.muniz.demiguel@usc.es (S.M.d.M.); franciscojavier.dieguez@usc.es (F.J.D.)

**Keywords:** ADHD-like, hyperactivity, behavior problems, dogs

## Abstract

**Simple Summary:**

Similar to humans, dogs could suffer a syndrome similar to Attention-Deficit/Hyperactivity Disorder (ADHD). Several studies suggest that dogs can serve as a model for studying ADHD. This condition leads to behavioral issues like impulsivity, attention problems, hyperactivity, and sometimes aggression, affecting both the dog’s and their caregiver’s quality of life. ADHD-like behavior in dogs is linked to disruptions in neurotransmitters like serotonin and dopamine, similar to humans. It seems to result from a mix of genetics and environment. In addition to the mentioned symptoms, dogs with ADHD-like behavior may also display compulsive behaviors, aggression, inappropriate elimination, fearfulness, epilepsy, foreign body ingestion, and itchiness. While there is no clear veterinary diagnosis for ADHD-like behavior in dogs, validated questionnaires can be helpful, but these cannot be used as a unique diagnostic tool. Treatment may involve medications like fluoxetine, along with environmental enrichment, relaxation techniques, and behavior modification to improve the dog’s and caregiver’s quality of life.

**Abstract:**

Similar to humans, dogs could suffer an Attention-Deficit/Hyperactivity Disorder-like syndrome (ADHD-like). In fact, several studies highlight the use of dogs as a model for studying ADHD. This condition entails behavioral problems expressed through impulsivity, attention issues, hyperactivity, and/or aggression, compromising the quality of life for both the caregiver and the dog. The pathophysiology of ADHD-like is complex and is associated with dysregulation of various neurotransmitters such as serotonin and dopamine. The expression of ADHD-like behavior in dogs would appear to depend on a classical gene–environment interaction as is the case with many neurological disorders in humans. In addition to the described symptomatology, ADHD-like dogs can exhibit strong comorbidities with compulsive behaviors, aggressiveness, inappropriate elimination and fearfulness, in addition to epilepsy, foreign body ingestion, and pruritus. In spite of the fact that there is no veterinary consensus about the diagnosis of ADHD-like, some validated questionnaires could be helpful, but these cannot be used as a unique diagnostic tool. The use of drugs, such as fluoxetine, in addition to an adequate environmental enrichment, relaxation protocols, and behavior modification can achieve an adequate quality of life for both the dog and caregivers.

## 1. Introduction

It is relatively common for a pet owner to seek out a behaviorist because their dogs exhibit excessive activity. Many times, this will be a case of excessive activity in a patient genetically predisposed to high energy levels (for example, working dogs), which are not being adequately stimulated or whose behaviors have been reinforced by the owner. Problems arise when the animal is denied the opportunity to exercise properly, deprived of necessary social interaction, and has poor control over its environment, so its behavior can appear ADHD-like.

On other occasions, excess activity may be associated with a more complex condition of Attention-Deficit/Hyperactivity Disorder (ADHD), similar to that in humans. This condition entails behavioral problems expressed through impulsivity, attention issues, hyperactivity, and/or aggression, compromising the quality of life for both the owner and the dog. Various authors have referred to this as hyperkinesis [1] or hypersensitivity–hyperactivity disorder [2,3]. Recently, the term ADHD-like has been proposed in the scientific literature to reflect its similarity with the disorder in children [4,5,6]. In this review, the term ADHD-like is used to refer to its presentation in canine species, and ADHD is used to refer to the presentation in humans.

Due to the similarities between ADHD-like in dogs and humans, several studies highlight the use of dogs as a model for ADHD [7,8,9,10,11]. ADHD is one of the most commonly diagnosed syndromes in pediatric psychiatry. Its most common symptoms, attention problems, hyperactivity, and impulsivity that interfere with everyday functioning in life, deteriorate relationships between children and their families and/or other children, increasing the risk of social isolation [12]. Furthermore, although ADHD is associated with childhood, two-thirds of diagnosed children continue to exhibit symptoms into adulthood [13,14]. Despite the fact that dogs could suffer from an ADHD-like syndrome, similar to ADHD in humans, the replicability of findings published in the field of ADHD-like in dogs is doubtful in many cases, given the absence of a unanimous definition and diagnostic criteria [1,2,4,5,7,9,15,16,17,18,19]. Additionally, methods used in human ADHD research are frequently absent, further contributing to uncertainty and the validity of the evidence concerning the existence of human analogue ADHD in dogs. In connection with this, it should also be pointed out that most research on dogs has only used ADHD-like traits as correlates for the investigation of various phenomena (for example, the investigation of correlates of questionnaire scores) [7,19,20], and not as a grouping variable (ADHD-affected vs. typical group), as used in human ADHD research. Furthermore, these studies employ questionnaires to assess the natural variability in these traits, indicating whether an individual is more or less attentive/active/impulsive. However, it is important to note that these scores do not necessarily imply an ADHD-like syndrome in dogs.

However, assessing the epidemiological situation of ADHD-like is challenging because of the variety of terminologies used in the literature. Epidemiological studies of behavioral problems in the canine population where excess activity is assessed—without determining its origin—showed figures ranging from 12 to 38.7% [21,22,23]. Similarly, Dinwoodie found a prevalence of 12% for hyperactivity/overactivity, scilicet dogs with impulsivity, overactivity, and high distractibility in an online questionnaire answered by pet owners [21]. Sulkama et al. found a 19.1% prevalence of inattention and 15% prevalence of hyperactivity/impulsivity. In their study, dog owners were asked to rate their dog’s behavior on a modified scale, developed by Vas et al. [24], which was designed to measure inattention, hyperactivity, and impulsivity [22]. The prevalence of hyperactivity in dogs referred to the Veterinary Behavior Medicine Service was 38.7%. Nevertheless, in this study only hyperactivity was defined as “dog moves constantly, moves fast, runs and jumps”, so it could encompass truly ADHD-like dogs without inattention, and high-energy normal dogs [23]. 

## 2. Pathogenesis

The pathophysiology of ADHD in humans is complex and is associated with dysregulation of various neurotransmitters such as serotonin, dopamine, norepinephrine, GABA, glutamate, and histamine, wherein cortical inhibitory processes that modulate subcortical excitatory activity are inadequate [25]. Several studies have suggested that the pathogenesis of ADHD-like behavior in dogs could be very similar and indeed propose dogs as a model for studying ADHD [17].

### 2.1. Human ADHD

ADHD in children is characterized by a decrease in the synthesis and an increase in the reuptake of dopamine and norepinephrine, ultimately resulting in problems in the regulatory circuits of various brain regions such as the prefrontal cortex, corpus callosum, and basal ganglia. In particular, children with ADHD have a smaller prefrontal cortex that operates at a slower pace under normal circumstances, influencing their ability to plan, correct errors, or avoid distractions. The globus pallidus and caudate nucleus are also smaller, leading to a reduced capacity to coordinate and filter information from other areas of the brain and inhibit automatic responses involved in impulse control. Overall, ADHD is associated with a total decrease in brain size of 3–5% [26] compared to control subjects, primarily due to a reduction in gray matter. In fact, consistently lower volumes have been documented in individuals with ADHD in the right globus pallidus, putamen, caudate nucleus, nucleus accumbens, amygdala, hippocampus, and cerebellum [27].

Changes in the brain throughout the lives of patients with ADHD are of great interest, as the prevalence of ADHD is age-dependent. Some volumetric alterations observed in childhood tend to normalize with age [13], suggesting that ADHD is related to a delay in the maturation of brain structures that typically mature at earlier ages in healthy controls and that brain maturation in ADHD is achieved at older ages [28].

The neurotransmitters implicated in ADHD in children, which may be extrapolated to cases of pathological hyperactivity in the canine species, are summarized in Table 1. Although dopamine appears to be the key neurotransmitter in this pathology, it has been observed that the paradoxically calming effects of psychostimulants, such as amphetamines, in patients with ADHD, are primarily due to their blockade of the dopamine transporter. They also block other monoamine transporters, resulting in an extracellular increase not only in dopamine but also in norepinephrine and serotonin [29].

### 2.2. The Role of Dopamine

Dopamine is involved in regulating motor activity and limbic functions but also plays a significant role in attention and cognition, especially in executive functions [30], motivation, and reward processing [31]. For example, children with ADHD require stronger incentives to modify their behavior than those without ADHD; they also show a failure to delay gratification, have impaired responses to partial schedules of reinforcement, and a preference for small immediate rewards over larger delayed rewards [49]. ADHD has been linked to dopamine dysfunction in mesocortical, mesolimbic [25], and nigrostriatal pathways [44]. Disturbances in the mesocortical dopamine pathway are thought to give rise to cognitive deficits. The mesocortical pathway has dopamine cell bodies in the ventral tegmental area (VTA) with projections via the medial forebrain bundle to the frontal cortex. As such, it acts as an important regulator of cognitive functioning. Disturbances in the mesolimbic pathway are thought to cause motivational deficits in ADHD. The mesolimbic dopamine pathway forms a crucial part of the brain’s “reward” circuits. Here, fibers from the VTA project via the medial forebrain bundle to discrete parts of the limbic system, especially to the nucleus accumbens [31]. Finally, the dopaminergic nigrostriatal pathway has projections from the substantia nigra to the striatum and plays a critical role in dopamine signaling [44], particularly, that involved in cognitive and voluntary movement control [50]. Some genetic markers relating dopamine to ADHD [12,50] and ADHD-like behavior have been identified. Polymorphisms in the tyrosine hydroxylase gene, an enzyme catalyzing dopamine precursor conversion, have been associated with impulsivity and hyperactivity [33]. Similar to humans, polymorphisms in the dopamine receptor gene, DRD4, have been associated with hyperactivity and impulsivity [51], as well as aggression [34], in dogs. Additionally, an association between polymorphisms in the dopamine beta-hydroxylase gene and the dopamine transporter gene was found in Belgian Shepherds with attention deficit [35].

**Figure 1:**
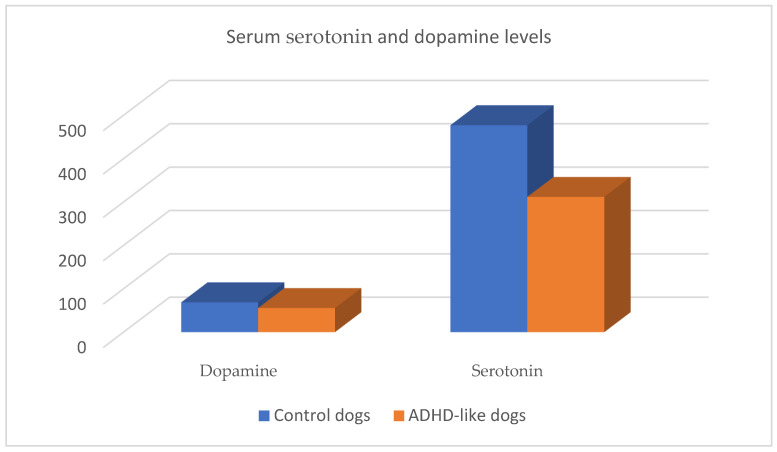
Serum serotonin and dopamine levels in control and ADHD-like dogs [9].

Moreover, low levels of dopamine were obtained in the urine of dogs with high impulsivity [43]. In a recent investigation, ADHD-like dogs were found to have significantly lower serum dopamine levels than control dogs (Figure 1). Similarly, dogs with higher hyperactivity and impulsivity scores had significantly less dopamine in their blood [9].

Further supporting the role of dopamine in ADHD pathogenesis is the use of methylphenidate as a treatment to reduce symptoms of this condition. Methylphenidate is a stimulant drug that inhibits dopamine reuptake in the synaptic cleft, thereby increasing its levels. It appears that approximately 70% of children and adolescents with ADHD respond well to methylphenidate [12,52]. Nevertheless, only some clinical cases were published in dogs. Luescher found a good response to methylphenidate in 3 out of 6 dogs [1], Piturru found a beneficial response in another case [15], though the treatment with methylphenidate had to be discontinued in the case published by Luño et al. [53].

### 2.3. The Role of Serotonin

The effects of serotonin on behavior are complex and nonspecific. Serotonin plays a significant role in mood regulation, appetite control, sleep, arousal, and pain regulation [54]. The monoaminergic systems in the brain do not act in isolation; they are all interconnected and interact reciprocally. For example, serotonergic neurons interact with neurons in the noradrenaline and dopamine systems [55]. Serotonin regulates dopamine and norepinephrine transmission through multiple serotonin receptors [29]. Serotonin action on 5HT1A receptors facilitates downstream norepinephrine and dopamine in the prefrontal cortex, a critical region for the regulation of attention and impulsive behavior. Nevertheless, serotonin binding at 5HT2C receptors on GABA interneurons inhibits norepinephrine and dopamine release [56]. Serotonergic disorders may also contribute to cognitive and behavioral impairments associated with ADHD, partly through its action on dopamine and norepinephrine. Different genetic polymorphisms that modulate the expression of serotonin transporters and receptors, which in turn regulate extracellular serotonin levels, have been found to increase susceptibility to ADHD [57]. Previous studies have found low blood serotonin levels in children with ADHD [37,58,59]. Chronic deficiency in available serotonin may contribute to the clinical symptoms of ADHD [60]. Serotonin neurotransmission can modulate the severity of symptoms of this disorder and has an impact on comorbidities, especially conduct disorder, obsessive–compulsive disorder, aggression, and affective disorders (major depression and/or anxiety) [37]. Thereby, drugs that inhibit serotonin reuptake pumps (SSRIs) are effective in both human medicine for treating ADHD [37] and veterinary medicine for treating ADHD-like behavior [2,16,53].

Neurocognitive models present inhibition as one of the main deficits in ADHD, which appears to be mediated by serotonin [37,61]. Several studies have investigated impulsivity and serotonin levels in dogs. Wright and colleagues found low serotonin and dopamine levels in urine samples of impulsive dogs [43], and similarly, low blood serotonin levels have been linked to impulsivity and aggression in dogs [39,40,41,42].

There are few studies published that measured serum serotonin levels in dogs. Dogs with ADHD-like behavior (vs. control dogs) showed significantly lower serotonin concentrations (Figure 1). Additionally, serotonin levels decreased in dogs with higher impulsivity, hyperactivity, and aggression scores [9].

### 2.4. The Role of Norepineprhyne

Norepinephrine is intimately linked to the dopaminergic system due to the fact that norepinephrine is a downstream product of dopamine metabolism. Norepinephrine neurotransmission significantly regulates higher cognitive functions, such as working memory and inhibitory control, although its projections primarily originate in the locus coeruleus and innervate multiple areas of the cortex, thalamus, and cerebellum. It has been suggested that innervation of the prefrontal cortex through norepinephrine pathways is particularly important for understanding ADHD. Norepinephrine and dopamine signaling are closely interconnected in the prefrontal cortex, meaning that they mutually influence the performance of cognitive tasks [62]. Atomoxetine, a selective norepinephrine reuptake inhibitor, is effective in treating the core symptoms of ADHD and some of its comorbidities. Similarly, many other prescription medications with noradrenergic properties, such as guanfacine and clonidine, are also effective [44,63]. Research conducted on norepinephrine and ADHD is less extensive than that on serotonin and dopamine, and no studies have been conducted in dogs.

### 2.5. Risk Factors Related to ADHD-like

Risk factors influencing the manifestation of ADHD-like behavior are comparable to those related to ADHD in humans [6]. The expression of ADHD-like behavior in dogs appears to depend on classical gene–environment interactions, as is the case with many neurological disorders in humans [4]. Furthermore, risk factors can be grouped into genetic, environmental, and demographic (Table 2). Risk factors can act as triggers or exacerbators in both physiological hyperactivity and neurotransmission problems.

#### 2.5.1. The Role of Genetics

The expression of ADHD-like behavior in dogs would appear to depend on a classical gene–environment interaction, as is the case with many neurological disorders in humans [4].

Previous studies detected significant differences in the tendency to be more excitable and require more activity. Hunting dogs and certain working breeds (e.g., herding dogs) have been selectively bred to perform tasks that require a high level of activity. The terrier group is characterized by traits such as high energy levels, excitement, reactivity, but also boldness [4] or inattention (Cairn Terrier, Golden Retriever, and Finnish Lapponian Dog) [6]. Nevertheless, these studies applied questionnaire-based methods, which only examine the occurrence and distribution of inattention, hyperactivity, and impulsivity traits but do not show whether these could be considered as diagnosable individuals.

Several studies found a relationship between ADHD-like and genetics. Sanders et al. [64] showed that energy levels in dogs were significantly associated with DNA methylation. Activity–impulsivity was associated with polymorphisms in the Tyrosine Hydroxylase (TH) gene [33] and polymorphisms in dopamine D4 receptor in German Shepherd dogs [35,36]. However, these studies do not claim that in dogs, certain genes and gene variants indicate pathological levels of activity; only lower/higher activity is associated with certain genes as a continuous variable. In a similar way, polymorphisms of the dopamine D4 receptor, dopamine-beta-hydroxylase, and dopamine transporter genes were associated with attention deficit in Belgian Tervuerens [51]. 

#### 2.5.2. The Role of the Environment

The absence of social play, especially in young individuals, the use of punishment or experiencing adverse events, the limited affiliative contacts (such as petting from caregivers), prolonged periods of separation after adoption (including sleeping away from caregivers), and spending extended periods alone were associated with the presentation of ADHD-like [4,6,20]. Rearing the puppy in a household with children may increase the tendency to be excitable, exhibit high energy levels, and show greater susceptibility to distractions [65]. Early weaning can also be considered a predisposing factor related to hyperactivity, as social contact deprivation during early development may lead puppies to engage in various excessive behaviors to make up for this lack [53,66]. Puppies isolated from littermates up to 12 weeks of age may exhibit deficits in social behavior, including aggression and hyperactivity [67].

Physical overstimulation, such as a high number of long walks that do not allow adequate rest time, was associated with ADHD-like in dogs [4]. Low physical exercise was also linked to increased hyperactivity/impulsivity [6].

#### 2.5.3. Demographic Risk Factors

A higher incidence of human ADHD has been reported in boys (compared to girls) [12,68]. However, inattentive presentation is more common in women, making it more difficult to detect [69]. Comparable results were found in dogs; ADHD-like prevalence is significantly higher in males [4,6,9]. Still, other studies did not find differences between males and females [7,8,19,70].

Some studies have suggested that entire animals were more prone to ADHD-like behaviors than castrated dogs [6,9]. Nevertheless, Zink et al. [71] found that neutering could be a risk factor for hyperactivity, whereas Fadel et al. [72] indicated that castrated animals showed higher impulsivity, and Csibra et al. [20] found that neutering dogs had a significatively higher tendency to show inattention and impulsivity. Though, owners might try to find a solution to deal with the condition of their dogs by neutering. Anyhow, the role of sex hormones in ADHD-like behaviors should be studied in more detail.

ADHD is a neurodevelopmental disorder whose onset typically occurs in childhood, between 6 and 12 years of age. Although symptoms tend to decrease with age (up to 65% of affected individuals experience partial remission), only 15% of diagnosed children present a complete remission of symptoms and functional improvement in early adulthood, characterizing ADHD as a chronic disorder [69]. Age was also a significant contributor to the prevalence of ADHD-like in some studies; therefore, hyperactive, impulsive, and inattentive behaviors are much more prevalent in young dogs [6,8,9,24]. This difference may be due to the fact that probably most adult dogs were treated and improved behavior. Nevertheless, there are no longitudinal studies on ADHD-like in dogs and their evolution, despite the fact that, as a neurodevelopmental disorder, this problem will affect the dog for life.

**Table 2:** Risk factors related to ADHD-like.

Risk Factors Related to ADHD-like
**Genetic factors**
Some breeds are more excitable and require more activity than others. Hunting dogs and some working dogs (for example, herding dogs) have been selected to be able to perform tasks that require a high level of activity. Terriers could have a predisposition due to their excitable, reactive, and energetic nature [4]. Cairn Terriers, Jack Russell Terriers, German Shepherds, and Staffordshire Bull Terriers seem more predisposed to hyperactivity/attention-deficit problems, while Cairn Terriers, Golden Retrievers, and Finnish Lapponian Dogs are more prone to attention deficits [6]. Some genes related to pathological excess activity have been identified, especially in German Shepherd and Belgian Shepherd dogs [33,34,35,36].
**Environmental factors**
Lack of social play, especially in young individuals. Use of punishment and experiencing adverse events. Limited affiliative contacts, such as petting, from caregivers. Prolonged periods of separation after adoption, including sleeping away from caregivers [4]. Spending an extensive amount of time alone [6]. Not participating in educational activities is associated with higher attention deficit [6]. Raising the puppy in a home with children may increase the tendency to be excitable, show high energy levels, and be more sensitive to distractions [65]. Early weaning [53,66]. Puppies isolated from littermates up to 12 weeks of age [67]. Physical overstimulation that does not allow adequate resting time. Low physical exercise was also linked to increased hyperactivity/impulsivity.
**Demographic factors**
Age: Young animals tend to be more active than adults. Sex: It appears to be more common in males [6,9]. Neutering in males, due to a lack of regulation caused by decreased testosterone [4,71], although some studies suggest otherwise [73].

## 3. Symptomatology

In humans with ADHD, three presentations have been described [68]:

- Concurrent hyperactivity/impulsivity and attention deficit.
- Predominantly inattentive presentation.
- Predominantly hyperactive/impulsive presentation.

Diagnosis entails a stipulation that symptoms persist for at least the preceding six months (Table 3), and there is clear evidence that the symptoms interfere with, or reduce, the quality of social, academic, or occupational functioning.

“Six (or more) of the following symptoms have persisted for at least 6 months to a degree that is inconsistent with developmental level and that negatively impacts directly on social and academic/occupational activities: Note: For older adolescents and adults (age 17 and older), at least five symptoms are required for each category. The symptoms are not solely a manifestation of oppositional behavior, defiance, hostility, or failure to understand task instructions”.

Analogous to human counterparts, dogs exhibiting ADHD-like behavior may not uniformly manifest all indicative signs, displaying profiles that are primarily impulsive/hyperactive, predominantly inattentive, or a blend of both. Table 4. Nonetheless, the manifestation of these behaviors should occur across varied contexts and with such frequency and intensity as to markedly impinge upon the quality of life of the dogs and their caretakers [2,4,9,16,51].

Some signs of hyperactivity/impulsivity described in the literature include difficulty in maintaining stillness, excessive vocalization, constant movement, persistent attention-seeking and play, prompt reaction or anticipation to events, impatience with waiting, less than 8 h of sleep a day [16] and poorer sleep quality [74], intolerance to delayed reward [43], excessive object destruction, lack of self-control (such as uninhibited biting), hypersensitivity (reaction to stimuli that are permanently present in the environment), lack of food satiety [16], and communication difficulties with other humans and dogs [2,5].

Additionally, tendency to be easily distracted, to lose interest easily, to demonstrate difficulty in maintaining concentration, to fail paying attention when directly addressed, to struggle with practical task execution, and to experience learning challenges are signs of inattention [8,75]. 

Furthermore, dogs with ADHD-like behavior may also exhibit somatic manifestations such as gastrointestinal signs, elevated heart and respiratory rates, dilated pupils, and increased temperature [1].

ADHD is frequently associated with comorbid aggression [50], anxiety, and phobias, consequences of neurotransmitter dysfunction [18,76,77]. Furthermore, ADHD-like dogs can exhibit strong comorbidities with compulsive behaviors, aggressiveness, and fearfulness [9,18,43]. Aggression is one of the comorbidities of being ADHD-like, precisely because of the tendency to behave impulsively, and it is also related to low serotonin levels. Serotonin is likely to mediate the tendency to behave aggressively and impulsively in ADHD-like dogs [9].

von Gontard et al. [78] reviewed the comorbidity of ADHD and incontinence in children and highlighted the importance of the association between these two conditions. Possible etiological and pathogenetic links between ADHD and incontinence have been provided by neurophysiological, imaging, and pharmacological studies. Nevertheless, no previous reports were published about house-soiling and ADHD-like in dogs. 

Clinical signs similar to those of ADHD have been reported in humans with epilepsy. Research findings indicate that approximately 12–15% of dogs with epilepsy exhibit elevated levels of hyperactivity/impulsivity [21,22], while approximately 20% of the general dog population showed signs of inattention [22,79].

Furthermore, regular shredding of objects associated with foreign body ingestion is mainly related to hyperactivity–impulsivity [80].

Interestingly, itch severity in dogs with atopic dermatitis (AD) is associated with an increased frequency of behaviors such as mounting, chewing, hyperactivity, stealing food, attention-seeking, excitability, excessive grooming, and reduced trainability [81]. A recent metanalysis found a significant association between AD and ADHD in humans [82]. AD and ADHD in humans share similar pathomechanisms involving inflammation, genetics, and microbiome. Consequently, several hypotheses have been proposed to illustrate the positive associations between AD and neurodevelopmental disorders [83].

**Table 4:** Signs related to ADHD-like.

Signs Related to ADHD-like in Dogs	
Hyperactivity–Impulsivity Signs	Attention Deficit Signs
Difficulty staying still. Excessive vocalization. Restlessness. Excessive attention demand and play. Higher reactivity/anticipation. Finds it hard to wait. Sleep less than 8 h a day [3] and poorer sleep quality [74]. Intolerance to delayed reward [70]. Lack of self-control (i.e., uninhibited biting). Excessive object destruction. Lack of satiety.	Any stimulus attracts attention. Easy loss of interest. Concentration difficulties. Lack of attention when someone is talking directly to them. Difficulty with practical tasks. Easily distracted. Learning problems.
**Somatic signs of ADHD-like [1]**	
Gastrointestinal signs, elevated heart and respiratory rates, dilated pupils, and elevated temperature.	
**Comorbidities related to ADHD-like [18,21,22,43,79,81]**	
Behavior problems: Compulsive disorders, aggression, phobias, house-soiling Medical problems: Epilepsy, foreign body ingestion, atopic dermatitis	

## 4. Diagnosis

ADHD-like cannot be diagnosed based on clinical signs alone, and no clear consensus for the diagnosis of canine hyperactivity or hyperkinesis syndrome or ADHD exists in either human or veterinary medicine [75]. As for the anamnesis, it is essential to conduct a thorough clinical history that includes a comprehensive ethological survey, gathering data on the environment, daily routine, or exercise level, to identify physiological and management characteristics that may justify the behavior. Key questions will include:

- Age at weaning and management.
- Triggering moments, locations, and times of the day when the behavior occurs.
- Relationship of the behavior with the presence or absence of the owner.
- Daily play and exercise activities of the animal.
- Times, duration, and contexts in which the dog is left alone or confined and social interactions.
- Daily resting time. Sleep behavior.
- Dog’s ability to learn commands and maintain interest in certain stimuli.
- Strategies used by the owner to curb or control the behavior.

Often, the excess of activity shown by a patient is not ADHD-like-related, but instead due to not pathologic increased energy, conditioned responses, inadvertent reinforcement, and unmet ethological needs (underexercise, and/or insufficient mental stimulation) [75]. In addition, similarly to other behavioral issues, various organic pathologies can cause hyperactivity, impulsivity, or attention-deficit behaviors. These may include metabolic derangements [84], CNS pathologies such as neoplasms, hydrocephalus or epilepsy with behavioral seizures, endocrine disorders like hypothyroidism, Cushing’s syndrome, or diabetes mellitus, hepatoencephalopathy; pathologies causing pain and discomfort in the animal; pathologies causing itching, cognitive dysfunction syndrome, lead poisoning, the use of certain medications that may, for instance, increase activity (as seen with corticosteroids), and anxiety disorders [75]. 

Physical, full neurological examination, complete blood count, biochemical panel, and urinalysis should be performed to rule out medical problems. Furthermore, thyroid panel and bile acids may be indicated when clinical alterations and analytical results suggest these conditions. Additional diagnostic tests, such as RX (in cases of suspected pain, gastrointestinal signs or foreign body ingestion), echography (in case of gastrointestinal signs, or foreign body ingestion), and TC or MRI (in case of alterations in neurological examination) should be performed based on the clinical signs observed.

In addition to the questionnaire typically used by the behaviorist in daily practice, the authors recommend using specialized and validated questionnaires such as the DIAS (Dog Impulsivity Assessment Scale) [43,70,85] and the ADHD Rating Scale for Dogs (Dog ARS) [3,8,19,24]. 

The DIAS questionnaire was developed to allow consistent assessment of impulsivity in dogs. It is an 18-item questionnaire which uses a 5-point Likert scale (scored 1–5) to rate the dog’s behavior. Thereby, the overall score of DIAS could provide an idea about the patient impulsivity; the reference values provided by the authors are the mean and the typical deviation [70]. The individual items comprising DIAS also cluster together, enabling the identification of three facets of impulsivity that span across behaviors, but may each be individually significant for patients with ADHD-like symptoms:

- Behavioral Regulation: a group of items reflecting excitability and ability to control both initiation and termination of behavior.
- Response to Novelty and Aggression: items indicating active responding when faced with aversion and uncertainty.
- Responsiveness: items describing behavioral sensitivity to environmental cues.

Although the DIAS questionnaire was not used for ADHD-like diagnoses, it could be helpful in defining impulsivity traits in ADHD-like dogs.

Vas et al. developed a 13-item questionnaire to measure inattention and hyperactivity/impulsivity. It was modified into a 12-item questionnaire and validated by Lit et al. [8]. It is a reliable tool for assessing ADHD-like behavior in dogs. However, despite a replication study indicating that it is not suitable for detecting diagnosable individuals, as it lacks items assessing functional impairment, the inclusion of owner-expert ratings in the evaluation process would be necessary [19]. Another previous study found that the Lit et al. 2010 version of the questionnaire had a very good ability to discriminate between ADHD-like dogs and healthy matched control dogs. Determining an appropriate threshold score can aid in screening for ADHD-like syndrome in dogs and prompt behavioral consultation. An initial working hypothesis suggests that a score of 29 or higher warrants a behavioral consultation. For scores between 24 and 29, a behavioral consultation is strongly recommended. Scores falling between 14 and 24 may indicate the need for behavioral modifications alone or implementation of a training program, accompanied by close monitoring. Scores below 14 make it unlikely that the dog has ADHD-like behavior, although this does not rule out the potential need for behavioral management. It is important to note that the Dog ARS should not be relied upon as the sole evaluation tool. A comprehensive behavioral assessment, including a differential diagnosis, must be conducted [3]. Nevertheless, it could be used as a helpful tool to assess ADHD-like, especially because it is the only questionnaire that was used to compare control and ADHD-like dogs.

A new promising tool is the questionnaire Dog ADHD and Functionality Rating Scale (DAFRS). It is a psychometrically improved scale to assess owners’ views on relevant dog behaviors with higher scores associated with greater functional impairment. DAFRS is a validated questionnaire; however, there are no studies that have used this questionnaire to differentiate ADHD-like dogs from control dogs, nor have they provided cut-off points for establishing a diagnosis [20,86].

Despite new studies suggesting variations in blood dopamine and serotonin levels in dogs with ADHD-like behavior [9], a diagnostic cut-off concentration has not been stablished. Therefore, blood analyses of serotonin and dopamine cannot be used for the diagnosis of ADHD-like.

Only in cases where there is a well-founded suspicion of an ADHD-like problem, meaning clinical criteria are met, and there is a suspicion of neurotransmission issues, would it be recommended to conduct a stimulant response test, in which case it would serve as the definitive diagnostic test. Hyperactive dogs are characterized by exhibiting a paradoxical response to treatment with amphetamines or methylphenidate [87]. These central nervous system stimulants increase dopamine levels by enhancing their release and blocking their reuptake, as well as that of noradrenaline. However, great caution must be exercised in conducting these tests, as the drugs can be diverted for human consumption, and there may be various difficulties in interpreting the results, such as inconsistent responses or lack of response, which are common [1,87]. For these reasons, this protocol is not validated for diagnostic of ADHD-like dogs [75].

There are various diagnosis criteria present in the research, and in many cases, the proposed behavioral signs are primarily based on observations and reports from dog owners, rather than on research with standardized methods and replicable protocols. The authors propose a diagnosis criterion for ADHD-like dogs summarized in Table 5. For ADHD-like diagnosis, the dog must meet all mandatory criteria. However, it is not necessary for dogs to exhibit all symptoms of hyperactivity, impulsivity, or attention deficit described in Table 4; they should display enough of them to obtain a score of ≥29 on the ARS questionnaire version by Lit et al.

## 5. Prognosis

Most dogs with ADHD show significant improvement [2,17]; many can be weaned off treatment, and the prognosis is generally good with proper treatment. However, the prognosis may be guarded if multiple comorbidities are present.

## 6. Treatment

If there is no medical pathology complicating the case, any instance of excessive activity or ADHD-like behavior warrants tailored behavior modification strategies. Additionally, some cases may require intervention with biological therapies or other tools. 

### 6.1. Behavior Modification Guidelines

No guidelines for behavioral modification have been published for ADHD-like. Also, no studies have been published showing the efficacy of several recommendations that can be found in the bibliography. Recommendations for any case of excess activity or ADHD-like behavior include:

- Avoiding situations that overstimulate the dog and trigger the behavior. It may be advisable for the owner to compile a list of stimuli or situations triggering the problematic behavior.
- Avoiding punishment, as it can lead to stress, fear, and other issues. In some cases, the need for activity may be so intense that even certain forms of punishment can be perceived as motivation [88,89].
- Withholding reinforcement from the owner, such as administering rewards at inappropriate times that may increase attention demands [90].
- Interacting with the dog when it is relaxed, ignoring exaggerated attention demands. If it becomes overly excited, it is advisable to use a signal to end the interaction, and if this fails, employ the “time out” technique (interrupting positive interaction) [90].
- Providing appropriate exercise for the breed and age of the animal [75,90,91].
- Maintaining routines regarding activities involving the animal to make its environment more predictable.
- Use of interactive and chew toys. A recent study suggests that chew toys provide relaxation during moments of stress for dogs [92]. Nevertheless, it should be monitored by pet parents because some dogs could become excessively excited or frustrated.
- Positive reinforcement training exercises, where quick rewards are recommended to maintain the animal’s interest, increasing dopamine in CNS.
- Implementing relaxation protocols [90,91]. The authors generally recommend practicing a relaxed down–stay on the dog’s bed. This exercise focuses on relaxed attention and impulse control to stay lying down.

### 6.2. Biological Therapies and Other Tools

If behavior modification strategies are not sufficiently effective or if any clinical signs require immediate control, the use of psychotropic drugs may be indicated.

The use of psychostimulants should be reserved only for confirmed cases of pathological hyperactivity where a positive response has been observed in the methylphenidate test. Interestingly, only few anecdotal cases were reported about the use of methylphenidate in ADHD-like dogs [1,15,53], and only three of out the six dogs treated by Luescher improved their condition [1]. Probably, methylphenidate is not the best choice for treating ADHD-like dogs.

As pharmacological alternatives, selegiline has been proposed for its action on dopamine, although it should not be used if aggression is present, and there are no studies about the use of selegiline in ADHD-like dogs. In humans, it did not yield better results than methylphenidate [93].

Clonidine, used in human medicine, may be used alone or in conjunction with methylphenidate for the treatment of patients with ADHD [94,95]. Although the use of clonidine in ADHD-like dogs has been suggested by some authors [96], there are no studies showing its effectiveness. Selective serotonin reuptake inhibitors (SSRIs) (fluvoxamine, sertraline, or fluoxetine) or tricyclic antidepressants (clomipramine) are generally recommended if aggression or impulsivity accompanies the condition. There is evidence supporting the beneficial use of fluoxetine in cases of hyperactivity, although it may need to be administered at higher doses than usual (2–4 mg/kg) [2,3]. Interestingly, fluoxetine blocks serotonin action at 5HT2C receptors and disinhibits (i.e., enhances) the release of both norepinephrine and dopamine [57]; therefore, it may be considered a first-line treatment for ADHD-like dogs. Another potentially interesting drug is venlafaxine; however, there are no scientific references to its use in this condition in dogs [97], and the results of its use in humans are controversial [63]. In cases where the patient experiences significant anxiety, combining serotonergic drugs with benzodiazepines may be a suitable option.

Recently, the effect of ketogenic diets on dogs with epilepsy and symptoms resembling ADHD has been studied, observing a reduction in behaviors such as predatory behavior and fear of unknown people. It is believed that the improvement in this case may be due to the potential anxiolytic effects of the diet [96].

In some instances, when walking is challenging or there are aggression issues, other tools may be necessary to facilitate dog control or reinforce safety measures, such as harnesses or muzzles, among others.

## 7. Prevention

Since ADHD-like is typically multifactorial, points to consider for preventing its onset should focus on controlling risk factors:

- Avoid breeding animals exhibiting signs of attention problems, hyperactivity, impulsivity, or aggression.
- Avoid early weaning of puppies to ensure adequate social experiences with their mother and littermates.
- Minimize prolonged periods of separation after the animal’s adoption.
- Fulfill the social needs of the animal through social play, petting, and other enjoyable interactions.
- Educate the dog in a positive manner.
- Provide appropriate exercise based on the age and breed of the animal.
- Provide conditions for the animal to sleep for enough hours.

## 8. Conclusions

In conclusion, understanding and addressing excessive activity or ADHD-like behavior in dogs requires a multifaceted approach that considers various factors, including genetics, environment, and neurobiology. While some cases may simply involve inadequate stimulation, others may reflect more complex conditions resembling ADHD in humans. To effectively manage these behaviors, thorough diagnostic evaluation, including behavioral assessments and differential diagnosis, is essential. Tailored behavior modification strategies, along with biological therapies, when necessary, can significantly improve outcomes. Additionally, preventive measures targeting risk factors such as breeding practices, socialization, and environmental enrichment are crucial for minimizing the development of ADHD-like behaviors in dogs. By integrating comprehensive diagnostic approaches with targeted interventions and preventive strategies, we can enhance the well-being and quality of life for both dogs and their caregivers. This paper could be a useful tool for a clinical approach to ADHD-like in dogs in the clinical practice and provides comprehensive information for future research.

## Figures and Tables

**Table 1:** The neurotransmitters involved in ADHD in children and, by similarity, in pathological excess activity in dogs.

Neurotransmitter	Role in ADHD
*Dopamine*	Participates in motor activity regulation and limbic functions, playing an essential role in attention and cognition, especially in executive functions [30] and reward processing [31]. In dogs, dopamine is associated with problems of aggression, impulsivity, attention deficits, and/or general nervousness [32,33,34,35,36].
*Serotonin*	Involved in mood and emotion regulation, and plays an important role in behavioral inhibition, which is affected in ADHD [37]. Altered serotonin transmission in patients with ADHD could be related to comorbidities with obsessive–compulsive disorders, aggression, and affective disorders (major depression and/or anxiety) [38]. In dogs, it is associated with aggression, impulsiveness, fear, and ADHD-like [9,39,40,41,42,43].
*Norepinephrine*	Norepinephrine neurotransmission plays an important role in regulating higher cognitive functions such as working memory and inhibitory control [44].
*Glutamate*	The most abundant excitatory neurotransmitter in the central nervous system. Involved in many neuronal functions such as synaptic transmission, neuronal migration, excitability, plasticity, impulsivity, and compulsivity [38]. Different genetic variations in the glutamatergic system have been associated with the presentation of ADHD symptoms [45,46].
*Histamine*	Regulates arousal and attention. Histaminergic neurons increase alertness and prevent sleep [47]. Plays an important role in neuro-immunological reactions. Children with ADHD are more likely to develop asthma, allergic rhinitis, atopic dermatitis, and allergic conjunctivitis than non-ADHD individuals [48].

**Table 3:** Signs related to ADHD in humans [68].

Signs Related to ADHD in Humans	
Inattention Signs	Hyperactivity and Impulsivity Signs
Often fails to give close attention to details or makes careless mistakes in schoolwork, at work, or during other activities (e.g., overlooks or misses details, work is inaccurate) Often has difficulty sustaining attention in tasks or play activities (e.g., has difficulty remaining focused during lectures, conversations, or lengthy reading)” Often does not seem to listen when spoken to directly (e.g., mind seems elsewhere, even in the absence of any obvious distraction) Often does not follow through on instructions and fails to finish schoolwork, chores, or duties in the workplace (e.g., starts tasks but quickly loses focus and is easily sidetracked)” Often has difficulty organizing tasks and activities (e.g., difficulty managing sequential tasks; difficulty keeping materials and belongings in order; messy, disorganized work; has poor time management; fails to meet deadlines)” Often avoids, dislikes, or is reluctant to engage in tasks that require sustained mental effort (e.g., schoolwork or homework; for older adolescents and adults, preparing reports, completing forms, reviewing lengthy papers)	*Hyperactivity*
	Often fidgets with hands or feet or squirms in seat Often leaves seat situations where remaining seated is expected (e.g., leaves his or her place in the classroom, in the office or other workplace, or in other situations that require remaining in place) Often runs about or climbs excessively in situations in which it is inappropriate (Note: In adolescents or adults, may be limited to subjective feelings of restlessness) Often unable to play or engage in leisure activities quietly Is often “on the go”, acting as if “driven by a motor” (e.g., is unable or uncomfortable being still for extended time, as in restaurants, meetings, etc.; may be experienced by others as being restless or difficult to keep up with)” Is often “on the go”, acting as if “driven by a motor” (e.g., is unable or uncomfortable being still for extended time, as in restaurants, meetings, etc.; may be experienced by others as being restless or difficult to keep up with)” Often talks excessively
	*Impulsivity*
	Often blurts out answers before questions have been completed (e.g., completes people’s sentences; cannot wait for turn in conversation) Often has difficulty awaiting turn (e.g., while waiting in line)” Often interrupts or intrudes on others (e.g., butts into conversations, games, or activities; may start using other people’s things without asking or receiving permission; for adolescents and adults, may intrude into or take over what others are doing)
**Several hyperactive–impulsive or inattentive symptoms that caused impairment were present prior to age 12 years**	
Several impairments from the symptoms are present in two or more settings (e.g., at home, school, or work; with friends or relatives; in other activities).	
There is clear evidence that the symptoms interfere with, or reduce, the quality of social, academic, or occupational functioning.	
The symptoms do not occur exclusively during the course of schizophrenia or another psychotic disorder and are not better accounted for by another mental disorder (e.g., mood disorder, anxiety disorder, dissociative disorder, or a personality disorder).	

**Table 5:** Proposed diagnosis criteria for ADHD-like.

Diagnosis Criteria for ADHD-like Dogs
**Compulsory criteria**
The behavior was present for at least 6 months. Signs of hyperactivity–impulsivity and/or attention deficit were present (Table 4), despite the fact that the dog’s ethological needs were satisfied. Medical problems are ruled out, or if they are present, do not explain the behavior. These behaviors should occur across varied contexts and with such frequency and intensity as to markedly impinge upon the quality of life of dogs and their caretakers. ARS questionnaire version of Lit et al. scores ≥ 29.
**Suspected criteria**
Somatic signs could be present, including gastrointestinal signs, elevated heart and respiratory rates, dilated pupils, and elevated temperature. DIAS results consistent with impulsivity. ARS questionnaire version of Lit et al. scores between 24 and 29. Behavioral comorbidities, in addition to signs of deficit attention and/or hyperactivity–impulsivity, include compulsive disorders, aggression, phobias, and house-soiling. Medical comorbidities include epilepsy, foreign body ingestion, and atopic dermatitis. Paradoxical response to treatment with amphetamines or methylphenidate.

## Data Availability

No new data were created.
